# Antimicrobial Peptide Epinecidin-1 Modulates MyD88 Protein Levels via the Proteasome Degradation Pathway

**DOI:** 10.3390/md15110362

**Published:** 2017-11-16

**Authors:** Bor-Chyuan Su, Jyh-Yih Chen

**Affiliations:** Marine Research Station, Institute of Cellular and Organismic Biology, Academia Sinica, 23-10 Dahuen Rd., Jiaushi, Ilan 262, Taiwan; su8265@gmail.com

**Keywords:** epinecidin-1, MyD88, A20, IRAK-M, SOCS-1, proteasome

## Abstract

The cationic antimicrobial peptide epinecidin-1 was identified from *Epinephelus coioides* and possesses multiple biological functions, including antibacterial, antifungal, anti-tumor, and immunomodulatory effects. In addition, epinecidin-1 suppresses lipopolysaccharide (LPS)-induced inflammation by neutralizing LPS and ameliorating LPS/Toll-like receptor (TLR)-4 internalization. However, it is unclear whether the actions of epinecidin-1 depend on the regulation of TLR adaptor protein MyD88 or endogenous TLR signaling antagonists, which include A20, interleukin-1 receptor associated kinase (IRAK)-M, and suppressor of cytokine signaling (SOCS)-1. Our results demonstrate that epinecidin-1 alone does not affect A20, IRAK-M, or SOCS-1 protein levels. However, pre-incubation of epinecidin-1 significantly inhibits LPS-induced upregulation of A20, IRAK-M, and SOCS-1. In addition, epinecidin-1 significantly reduces the abundance of MyD88 protein. Both MG132 (a specific proteasome inhibitor) and Heclin (a specific Smurf E3 ligase inhibitor) are able to abolish epinecidin-1-mediated MyD88 degradation. Thus, our data suggest that epinecidin-1 directly inhibits MyD88 via induction of the Smurf E3 ligase proteasome pathway.

## 1. Introduction

Toll-like receptor (TLR) signaling is crucial for modulating innate and adaptive immune responses in physiological and pathological conditions [1]. Lipopolysaccharide (LPS) from Gram negative bacteria can bind to TLR4 [2], and as such, this receptor can detect invasion of either intact bacterial cells or their components [1]. Furthermore, the activation of TLR4 signaling is required to suppress Gram negative bacterial infection [3]. Upon TLR4 activation, TLR adaptor protein MyD88, interleukin-1 receptor-associated kinase (IRAK)-1, IRAK-4, and tumor necrosis factor receptor-associated factor (TRAF)-6 form a TLR signaling complex, which in turn activates mitogen activated protein kinase (MAPK) and nuclear factor-κB (NF-κB) signaling [4,5]. NF-κB then mediates the production of inflammatory cytokines [6], which regulate diverse immunological activities to inhibit the spread and growth of pathogens [7]. For instance, tumor necrosis factor (TNF)-α gene expression is induced by NF-κB and serves to not only facilitate macrophage recruitment to lesions, but also enhancs phagocytosis of bacteria by macrophages [8,9]. While TLR-mediated inflammatory responses are essential for the elimination of pathogens [1], over-activation of TLR signaling may contribute to the etiology of inflammatory disorders, such as inflammatory bowel diseases [10], chronic inflammation [11], cytokine storm [12], and autoimmune diseases [13]. Endogenous TLR signaling antagonists can serve as signaling brakes to prevent the over-activation of TLR-signaling. These antagonists include zinc finger protein A20, IRAK-M, suppressor of cytokine signaling (SOCS)-1, and others [14]. Among these inhibitors, A20 suppresses TLR signaling by triggering TRAF-6 degradation [15], while IRAK-M inhibits the dissociation of IRAK-1 and IRAK-4 from the TLR/IRAK-1/IRAK-4 signaling complex [14]. SOCS-1 blocks TLR signaling by binding to IRAK-1 and regulating its activity [16]. Upregulation of these endogenous TLR signaling antagonists can suppress LPS- and TNF-α-induced inflammation [17,18].

*Epinephelus coioides* (orange-spotted grouper) is a warm water and seawater fish [19], which is found in turbid coastal reefs between the western Indian Ocean and the western Pacific Ocean [20]. Epinecidin-1, a cationic antimicrobial peptide, was identified from orange-spotted grouper [21] and possesses diverse biological activities, including antibacterial, antifungal, anti-cancer, and immunomodulatory actions [22,23,24]. We previously demonstrated the therapeutic effect of epinecidin-1 on LPS-induced endotoxemia [25]. In that study, we found that epinecidin-1 directly interacts with LPS in vitro and prevents its interaction with LPS binding protein. This action suppresses TLR4 endocytosis, thereby attenuating LPS-induced accumulation of reactive oxygen species, activation of p38, Akt and NF-κB, and subsequent proinflammatory cytokine production [25]. However, the direct binding to LPS and block on TLR4 activation cannot explain many of the other varied effects that have been observed after epinecidin-1 treatment. Therefore, we wanted to test the regulatory effects of epinecidin-1 on molecules with a broader range of physiological actions. Since canonical MyD88/NF-κB signaling responds to and controls many of the biological functions that epinecidin-1 is known to influence, we suspected that epinecidin-1 might antagonize key components of this pathway. Thus, we examined the effects of epinecidin-1 on three well-characterized endogenous TLR signaling antagonists (A20, IRAK-M, and SOCS-1) and the critical TLR signaling adaptor protein, MyD88, in Raw264.7 mouse macrophage cells.

## 2. Results

### 2.1. LPS Elevates the A20, IRAK-M and SOCS-1 Protein Levels in Raw264.7 Macrophages

**Figure 1:**
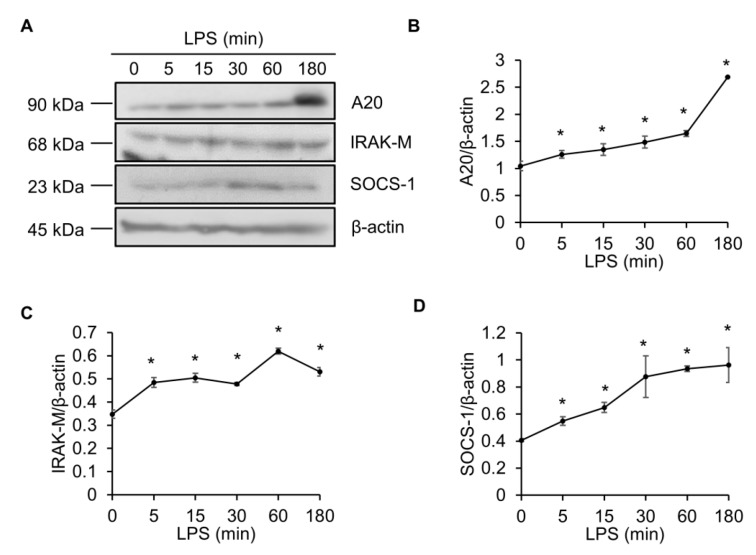
Lipopolysaccharide (LPS) elevates A20, IRAK-M and SOCS-1. (**A**) Raw264.7 cells were treated with LPS (100 ng/mL) for 0, 5, 15, 30, 60, or 180 min. Cell lysates were immunoblotted with A20, IRAK-M, and SOCS-1 antibodies, and band intensities were analyzed with ImageJ. Quantification of results for A20 (**B**), IRAK-M (**C**), and SOCS-1 (**D**). β-actin served as a loading control. * *p* < 0.05 versus 0 min.

To determine whether the protein levels of endogenous TLR signaling antagonists were increased upon LPS stimulation, Raw264.7 macrophage cells were treated with LPS for different times, and the protein levels of A20, IRAK-M, and SOCS-1 were probed by western blotting (Figure 1). The blots showed that A20 was slightly upregulated at the beginning of LPS treatment, followed by a robust induction at 3 h (Figure 1A,B). IRAK-M and SOCS-1 were also induced by LPS (Figure 1A,C,D). These results demonstrated that LPS treatment upregulates the endogenous TLR signaling inhibitors, including A20, IRAK-M, and SOCS-1.

### 2.2. Epinecidin-1 Alone Does Not Affect the A20, IRAK-M and SOCS-1 Protein Levels

**Figure 2:**
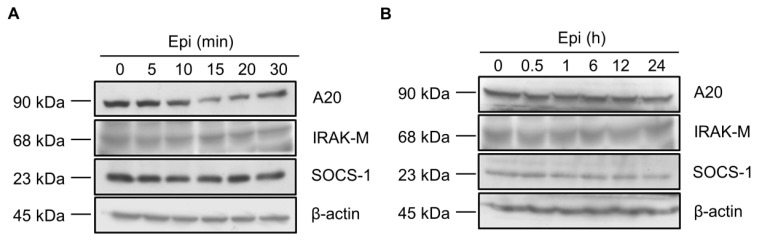
Epinecidin-1 alone does not affect protein levels of A20, IRAK-M, and SOCS-1. (**A**,**B**) Raw264.7 cells were incubated with epinecidin-1 (Epi; 6 μg/mL) for short-term (0–30 min) or long-term (0–24 h) exposure. After treatment, cell lysates were collected and immunoblotted with indicated antibodies. β-actin served as a loading control.

**Figure 3:**
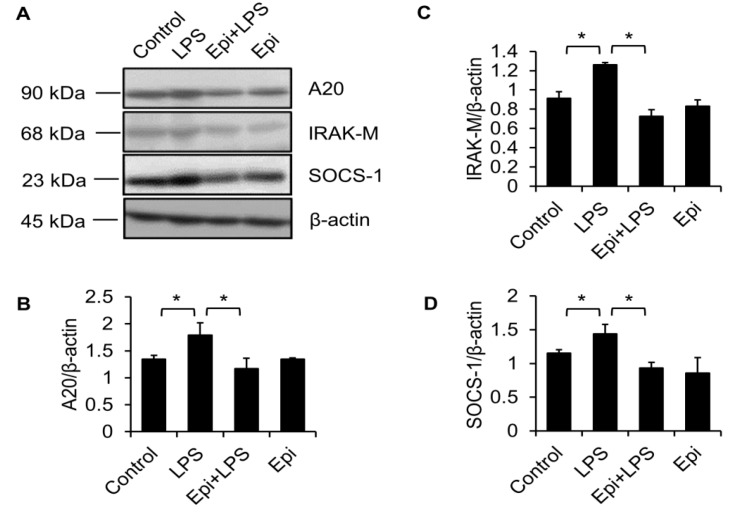
Epinecidin-1 attenuates LPS-induced upregulation of A20, IRAK-M, and SOCS-1. (**A**) Cells were preincubated with Epi (6 μg/mL) for 30 min, followed by treatment with LPS (100 ng/mL) for additional 30 min. Cell lysates were collected after stimulation with Epi/LPS and probed with indicated antibodies. β-actin served as a loading control. Quantification of results for A20 (**B**), IRAK-M (**C**), and SOCS-1 (**D**). * *p* < 0.05 versus LPS only.

To test whether epinecidin-1 directly regulates the levels of endogenous TLR signaling antagonists, Raw264.7 cells were treated with epinecidin-1 for different times, as indicated. Results showed that protein levels of A20, IRAK-M, and SOCS-1 were not altered either by short-term (Figure 2A) or long-term (Figure 2B) exposure of Raw264.7 cells to epinecidin-1. Next, cells were pre-incubated with epinecidin-1 for 30 min, followed by LPS treatment for an additional 30 min. We found that LPS-induced upregulation of A20 (Figure 3A,B), IRAK-M (Figure 3A,C), and SOCS-1 (Figure 3A,D) were attenuated by epinecidin-1. These results suggest that epinecidin-1 does not directly regulate the abundance of A20, IRAK-M, and SOCS-1, however, pre-incubation of epinecidin-1 can blunt LPS-induced upregulation of endogenous TLRs signaling antagonists.

### 2.3. LPS Upregulates MyD88 Protein Level

**Figure 4:**
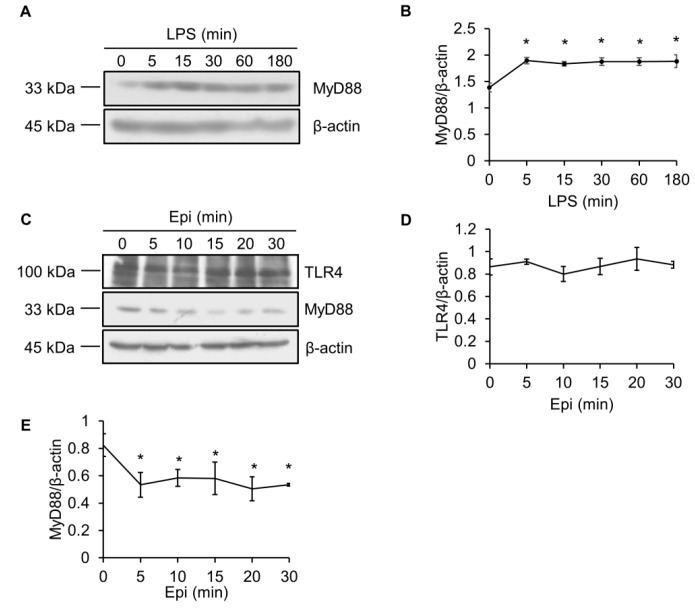
Epinecidin-1 suppresses protein levels of MyD88. (**A**) Cells were treated with LPS (100 ng/mL) for 0, 5, 15, 30, 60, or 180 min, and lysates were immunoblotted with MyD88 and β-actin. (**B**) Quantification of MyD88 protein levels. (**C**) Cells were treated with 6 μg/mL Epi for 0, 5, 10, 15, 20, or 30 min. Cell lysates were harvested after stimulation and immunoblotted with indicated antibodies. Quantification of TLR4 (**D**) and MyD88 (**E**) levels. * *p* < 0.05 versus 0 min.

Since MyD88 is a crucial adaptor protein that is essential for TLR signaling [26], we also determined the effect of epinecidin-1 and LPS on MyD88 levels. To test whether the abundance of MyD88 protein was modulated by LPS, Raw264.7 cells were treated with LPS for different times, as indicated. We found that MyD88 quickly (within 5 min) accumulated after LPS treatment (Figure 4A,B).

### 2.4. Epinecidin-1 Downregulates MyD88 Protein Level

**Figure 5:**
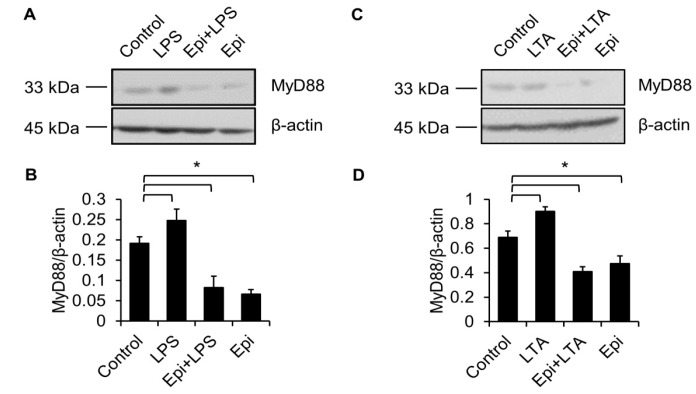
LPS-induced upregulation of MyD88 is abolished by epinecidin-1. Cells were preincubated with Epi (6 μg/mL) for 30 min followed by treatment with 100 ng/mL LPS (**A**) or 5 μg/mL lipoteichoic acid (LTA; **C**) for an additional 30 min. Cell lysates were collected and probed with MyD88 and β-actin antibodies. (**B**,**D**) Quantification of MyD88 level. * *p* < 0.05.

TLR4 is essential for LPS recognition [12], so we investigated the effect of epinecidin-1 on TLR4 protein levels. We found that epinecidin-1 did not affect the abundance of TLR4 (Figure 4C,D). Next, to address whether MyD88 is directly regulated by epinecidin-1, Raw264.7 cells were stimulated with epinecidin-1 for different times. Interestingly, we found that MyD88 was downregulated by epinecidin-1 in a time dependent manner (Figure 4C,E). In addition, pre-incubation of Raw264.7 cells with epinecidin-1 for 30 min significantly reduced the LPS-induced upregulation of MyD88 (Figure 5A,B). Because MyD88 is also required for the response of other TLRs to ligand binding, such as lipoteichoic acid (LTA) binding to TLR2 [27], we further tested the effects of LTA in combination with epinecidin-1 on MyD88. Results show that MyD88 was slightly increased upon LTA stimulation, and that this LTA-induced upregulation of MyD88 abundance was abolished by epinecidin-1 pretreatment (Figure 5C,D).

### 2.5. Proteasome Degradation Pathway Is Required for Epinecidin-1-Mediated MyD88 Downregulation

**Figure 6:**
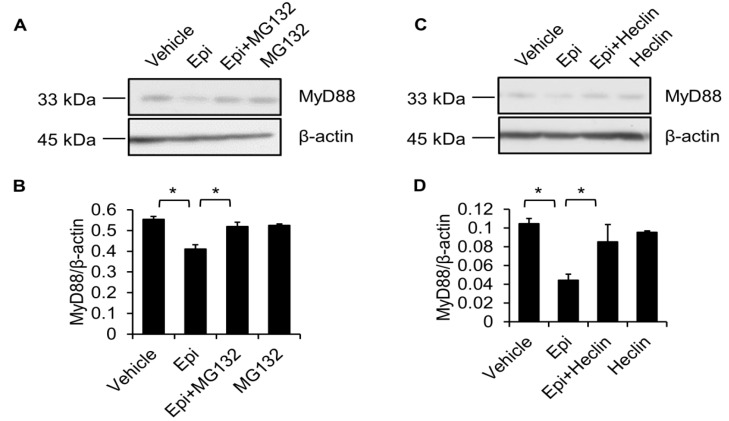
MG132 and Heclin abolish epinecidin-1-mediated degradation of MyD88. Cells were preincubated with 10 μM MG132 (**A**) or 7 μM Heclin (**C**) for 1 h followed by treatment with 6 μg/mL Epi (**A**) or for an additional 30 min. Cell lysates were collected and probed with MyD88 and β-actin antibodies. (**B**,**D**) Quantification of MyD88 levels. Vehicle: 0.5% DMSO. * *p* < 0.05.

The proteasomal degradation pathway is a well-known and major mechanism of regulating protein homeostasis [28]. Therefore, we tested whether this pathway is involved in epinecidin-1-induced MyD88 degradation. To test this possibility, Raw264.7 cells were pre-incubated with a specific proteasome inhibitor, MG132, followed by treatment with epinecidin-1 for 30 min. The results show that MG132 significantly attenuated epinecidin-1-mediated MyD88 degradation (Figure 6A,B). A previous study demonstrated that Smurf E3 ligase is involved in TGF-β-mediated MyD88 degradation [29]. To test whether Smurf E3 ligase is also involved in epinecidin-1-induced MyD88 degradation, cells were pre-incubated with a specific Smurf E3 ligase inhibitor, Heclin. Pretreatment with Heclin ameliorated epinecidin-1-mediated MyD88 degradation (Figure 6C,D). These results demonstrate that epinecidin-1-induced MyD88 degradation is dependent on Smurf E3 ligase and the proteasomal degradation pathway.

## 3. Discussion

Previous studies have demonstrated that upon LPS binding to TLR4, A20 is quickly induced and acts as a negative regulator of TLR4 signaling [30]. In this capacity, A20 induces the degradation of TRAF-6 or inhibits the phosphorylation of the IκB kinase complex to suppress NF-κB activation [31,32]. In addition to TLR4, A20 also negatively regulates TLR2- and TLR5-mediated signaling [33]. A20 knockout mice develop excessive multi-organ inflammation and are hypersensitive to LPS, which results in premature death [34], while hepatocyte-specific A20 knockout mice exhibit a greater susceptibility to LPS-induced liver inflammation than wild type mice [34]. In addition, A20-overexpressing mice are resistant to intracerebral hemorrhage-induced inflammatory injury [35]. Along with A20, IRAK-M also serves as a key negative regulator of TLR signaling by blocking the dissociation of IRAK-1, and -4 from the TLR/IRAK-1/IRAK-4 signaling complex, an action that inhibits TLRs signaling [32]. Several TLR ligands are able to upregulate the protein levels of IRAK-M, including LPS [36], LTA [37], and flagellin [38]. Moreover, IRAK-M knockout mice display a relatively strong inflammatory response upon bacterial challenge, suggesting that this protein is crucial for physiological control of inflammation [39]. Similar to IRAK-M and A20, SOCS-1 is induced by several pro-inflammatory signals, including LPS [40], LTA [41], and bacterial CpG-DNA [41]. SOCS-1 negatively regulates TLR signaling by facilitating MyD88-adaptor like protein degradation or interacting with IRAK-1 [41]. Furthermore, SOCS-1 blocks NF-κB activation by binding to the p65 subunit of NF-κB and enhancing p65 degradation [42]. Overexpression of SOCS-1 has been shown to suppress LPS-induced NF-κB activation and inflammation [40]. Thus, A20, IRAK-M, and SOCS-1 are all physiologically important factors that negatively regulate different TLRs-mediated signaling events and inflammation.

In this study, we found that epinecidin-1 treatment alone did not alter the protein levels of A20, IRAK-M, or SOCS-1, whereas the pre-incubation of epinecidin-1 blocked LPS-induced upregulation of these endogenous TLRs signaling antagonists. These results suggest that epinecidin-1 indirectly regulates endogenous TLRs signaling antagonists. We previously demonstrated that epinecidin-1 suppresses LPS-mediated signaling in the extracellular milieu [25] by directly interacting with LPS to prevent LPS-LPS binding protein interactions, LPS-TLR4 internalization, and subsequent inflammatory response [25]. Therefore, we postulate that epinecidin-1 blocks LPS-induced induction of A20, IRAK-M and SOCS-1 as a result of its ability to neutralize LPS.

We also demonstrated that degradation of MyD88 was induced by epinecidin-1, and we further showed that MG132 and Heclin significantly blunted epinecidin-1-mediated degradation of MyD88. Smurf E3 ligase is known to polyubiquitinate several target proteins [29], after which the ubiquitinated targets may be recognized by the proteasome for degradation [28]. We have shown for the first time that epinecidin-1 facilitates the degradation of MyD88 protein via Smurf E3 ligase and the proteasomal degradation pathway. MyD88 is a general TLR adaptor protein that mediates the responses of all of the TLRs, except for TLR3 [33]. The importance of this protein is demonstrated by MyD88 knockout mice, which show defective response to LPS [43]. Thus, MyD88 is considered to be a promising therapeutic target for inflammatory diseases [44]. Pharmacological inhibition of MyD88 was found to protect against renal injury induced by ischemia-reperfusion, both in vivo and in vitro [45], and MyD88 inhibitory peptide was shown to suppress adenovirus type 37-induced keratitis [46]. In addition to mediating signal transduction from TLR ligands, MyD88 is also required for signaling events and cellular activities that are induced by other cytokines or compounds. For instance, MyD88 is critical for Interleukin (IL)-1 and IL-18 receptor signaling [47]. Because of this action, IL-1-mediated T cell proliferation is abolished in MyD88 knockout mice [48]. IL-18-mediated natural killer cell activation and interferon-γ production were also found to be impaired in MyD88 knockout mice [47,49]. Furthermore, MyD88 is essential for Taxol-induced cytotoxicity in human myelomonocytic cells, lung cancer A549 cells and human epithelial ovarian carcinoma cells [50]. A previous study also demonstrated that concanavalin A-induced hepatitis requires MyD88-dependent signaling [51]. Thus, MyD88 plays a crucial role in a diverse set of pathophysiological states and pharmacological actions. In this study, we found that the degradation of MyD88 was induced by epinecidin-1, suggesting a potential therapeutic application for epinecidin-1 in MyD88-related diseases. Endotoxemia may occur in patients with uncontrolled bacterial infections, potentially causing multiple organ failure and death [52]. We recently demonstrated the therapeutic potential of epinecidin-1 in murine models of LPS-induced endotoxemia. In these models, we found that epinecidin-1 acts, at least partially, by disrupting the interaction between LPS and LPS binding protein [25]. In the current study, we demonstrate another potential mechanism that may contribute to epinecidin-1-mediated anti-inflammatory effects. Here, we show that MyD88 degradation is induced by epinecidin-1 via Smurf E3 ligase and the proteasome pathway, suggesting that epinecidin-1 may act at multiple levels to regulate LPS-induced TLR signaling.

To date, little is known about how antimicrobial peptides may regulate the proteasomal degradation pathway. However, a few relevant cases have been previously reported. For example, PR39, a proline and arginine-rich peptide, was identified from pig intestine and was shown to bind to the 26S proteasome [53]. This binding inhibited the proteasomal degradation of NF-κB inhibitor IκBα and caused suppression of TLR signaling [53]. Another example of a proteasome-modulating antimicrobial peptide is Baceridin, which was identified from the culture supernatant of a plant-associated *Bacillus* strain [54]. This peptide can block proteasome activity and inhibits cell cycle progression in multiple cancer cell lines [54]. Interestingly, both PR39 and Baceridin were shown to be proteasome inhibitors, whereas epinecidin-1 seems to be a proteasome activator. Epinecidin-1 induces the degradation of MyD88 via Smurf E3 ligase, however, the mechanism by which Smurf E3 ligase is activated by epinecidin-1 still requires further investigation.

**Figure 7:**
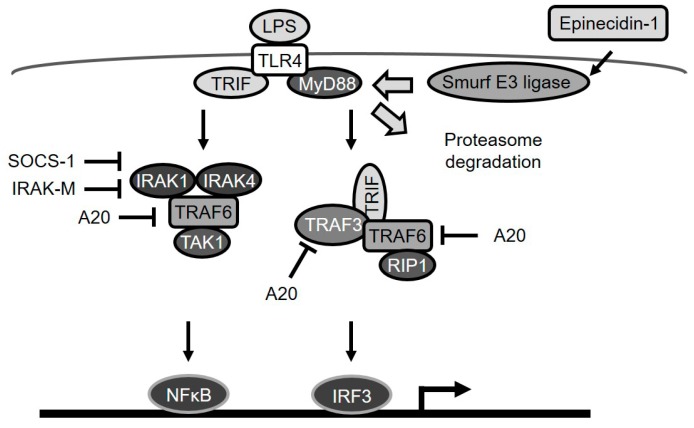
A diagram depicting epinecidin-1-mediated degradation of MyD88a and downstream events. LPS/TLR4 signaling is transmitted through MyD88-dependent or MyD88-independent pathways. A20 blocks TRAF6 and TRAF3. IRAK-M, and SOCS-1 suppress IRAK-1 and -4. Epinecidin-1 activates Smurf E3 ligase, which in turn facilitates MyD88 degradation by the proteasome.

Taken together, our data show that epinecidin-1 induces the degradation of the TLR signaling adaptor protein, MyD88, through the activation of Smurf E3 ligase and the proteasome degradation pathway (Figure 7). As a probable consequence of this MyD88 regulation, LPS-induced upregulation of endogenous TLR signaling antagonists (A20, IRAK-M, and SOCS-1) was also found to be abolished by epinecidin-1.

## 4. Material and Methods

### 4.1. Reagents

Epinecidin-1 (GFIFHIIKGLFHAGKMIHGLV-NH2; Epi) peptide was purchased from GL Biochem (Shanghai, China). Lipopolysaccharide (LPS) from *Escherichia coli* O111:B4, Lipoteichoic acid (LTA) from *Staphylococcus aureus*, MG132 and Heclin were purchased from Sigma. Epi was dissolved in dissolved in normal saline. LPS and LTA were dissolved in distilled water. MG132 and Heclin were dissolved in DMSO.

### 4.2. Cell Culture and Treatment

Raw264.7 (mouse macrophage) cells were purchased from the Bioresource Collection and Research Center (Hsinchu, Taiwan). Cells were maintained in the Dulbecco’s Modified Eagle’s medium (Gibco) with 10% fetal bovine serum (Gibco) and penicillin-streptomycin (Gibco) at 37 °C in a humidified environment with 5% CO_2_. To evaluate whether epinecidin-1 and LPS regulate TLR antagonists, MyD88 and TLR4, cells were treated with epinecidin-1 (6 μg/mL), LPS (100 ng/mL), or LTA (5 μg/mL) for various times (indicated in figure legends). To determine whether the proteasome pathway is involved in epinecidin-1-mediated MyD88 degradation, cells were pretreated with a specific proteasome inhibitor MG132 (10 μM) or Smurf E3 ligase inhibitor Heclin (7 μM) for 1 h, followed by treatments with epinecidin-1 (6 μg/mL) for additional 30 min.

### 4.3. Western Blotting Analysis

After treatment, cell lysates were collected in RIPA lysis buffer (Merck, Darmstadt, Germany) and were immunoblotted with following antibodies: A20 (Santa Cruz, TX, USA), IRAK-M (Millipore, Darmstadt, Germany), β-actin (Cell Signaling Technology, Danvers, MA, USA), SOCS-1 (Cell Signaling Technology), MyD88 (Cell Signaling Technology), and TLR4 (Cell Signaling Technology). Band intensity of western blots was analyzed with ImageJ (1.51j8).

### 4.4. Statistical Analysis

Data are expressed as mean ± SEM. Significance was determined by Student’s *t* test, and one-way ANOVA. *p* < 0.05 was considered statistically significant.
